# Macroangiopathy is a positive predictive factor for response to immunotherapy

**DOI:** 10.1038/s41598-019-46189-6

**Published:** 2019-07-05

**Authors:** Katerina Deike-Hofmann, Lukas Gutzweiler, Julia Reuter, Daniel Paech, Jessica C. Hassel, Oliver Sedlaczek, Alexander Radbruch, Heinz-Peter Schlemmer, Philipp Bäumer

**Affiliations:** 1 Department of Radiology, German Cancer Research Center (DKFZ), Heidelberg, Germany; 2 Department of Dermatology, National Center for Tumor Diseases (NCT), Heidelberg, Germany; 3 Department of Radiology and Neuroradiology, University Hospital Essen, Essen, Germany

**Keywords:** Melanoma, Predictive markers, Predictive markers

## Abstract

Immunotherapies demand for predictive biomarkers to avoid unnecessary adverse effects and costs. Analytic morphomics is the technique to use body composition measures as imaging biomarkers for underlying pathophysiology to predict prognosis or outcome to therapy. We investigated different body composition measures to predict response to immunotherapy. This IRB approved retrospective analysis encompassed 147 patients with ipilimumab therapy. Degree of macroangiopathy was quantified with the newly defined total plaque index (TPI), i.e. the body height corrected sum of the soft and hard plaque volume of the infrarenal aorta on portalvenous CT scans. Furthermore, mean psoas density (MPD), different adipose tissue parameters as well as degree of cerebral microangiopathy were extracted from the imaging data. Subsequent multivariate Cox regression analysis encompassed TPI, MPD, serum LDH, S100B, age, gender, number of immunotherapy cycles as well as extent of distant metastases. TPI and MPD correlated positively with PFS in multivariate analysis (p = 0.03 and p = 0.001, respectively). Furthermore, single visceral organ and/or soft tissue involvement significantly decreased progression risk (p = 0.01), whereas increased S100B level showed a trend towards PFS shortening (p = 0.05). In conclusion, degree of macroangiopathy and sarcopenia were independent predictors for outcome to immunotherapy and of equivalent significance compared to other clinical biomarkers.

## Introduction

Immune checkpoint inhibitors have yielded promising clinical responses in patients with advanced malignant melanoma^1–6^. However, as objective response rates are highly variable^7^, it is crucial to define patients that are likely to benefit from immune checkpoint blockade to avoid unnecessary adverse effects and costs.

Analytic morphomics is the technique to use body composition measures as imaging biomarkers for underlying (patho)physiology to predict prognosis or outcome to therapy. Imaging surrogates of sarcopenia such as the mean density of the psoas muscle (MPD) have repeatedly been shown to correlate with post-interventional outcome^8–16^. As non-specific immunotherapies such as the CTLA-4 antibody ipilimumab do not directly target the tumor but augment the pre-existing, but clinically insufficient body-inherent immune system to fight cancer, the conventional pharmacodynamic model applied to standard chemotherapeutics is not completely applicable. Therefore, it seems reasonable that the biological and immunological fitness of the host may be a decisive prerequisite for response to immunotherapy.

Previous morphomics studies focused on conventional therapies such as surgery, radio- or chemotherapy and the few which investigated the correlation of morphomics and outcome after immunotherapy again concentrated on the sarcopenia-frailty-concept^17–19^. Therefore, we sought to investigate the potential of morphomics to identify surrogates of the immune status beyond tissue parameters proposed in the present literature. We hypothesized that vascular health might influence outcome to immunotherapy simply because the vascular status might be a surrogate of fitness, which in turn might influence outcome to immunotherapy as the effect of immunotherapies depends on host-inherent resources, i.e. the immune system of the patient, to fight cancer.

Furthermore, macro- and microangiopathy are known to be systemic inflammatory diseases associated with endothelial activation and – dysfunction as well as increased levels of pro-inflammatory chemo- and cytokines. Consecutive inappropriate regulation of the vascular tone, permeability, coagulation, fibrinolysis as well as cell adhesion and proliferation might hypothetically interact with effects of immunotherapy^20–22^.

Primary aim of this study was to investigate the association between vessel health in terms of imaging signs of macro- and microangiopathy and progression free survival (PFS) after immune therapy with the CTLA-4 antibody ipilimumab. As secondary aims we sought to compare this potential effect with other body composition measures as well as further clinical and blood-based parameters.

## Results

### Patient data

Overall, 147 melanoma patients were included, 57 female (38.8%) and 90 male (61.2%). Patients’ median age (interquartile range (IQR)) was 60.0 (49.5–66.5) years and median number of received immunotherapy cycles was 4 (range 1–4). Patients who received a total of four cycles of ipilimumab therapy (n = 89, 60.5%) showed a trend towards increased PFS (univariate Cox regression analysis, p_univ_ = 0.18, hazard ratio (HR) 0.79, 95% confidence interval (CI) 0.55–1.12).

50 of 147 patients (34.7%) displayed distant metastases in more than one visceral organ (with or without nodal or cutaneous involvement^23^), which showed close to significant association with an increased risk for disease progression in univariate analysis (p_univ_ = 0.07, HR 1.39, CI 0.97–2.00).

Until the end of the study, 128 patients (87.1%) had shown progressive disease, while 19 (12.9%) were stable. Median time to progress (IQR) was 124.9 (14.8–164.0) days. Detailed patient characteristics are shown in Table 1.

**Table 1:** Patient characteristics.

Parameter	Total			Women			Men		
	n	Median	IQR	n	Median	IQR	n	Median	IQR
**Clinical parameter**									
Age [years]	147	60	49.5–66.5	57	57	49.0–63.0	90	62	50.0–69.0
Weight [kg]	144	78	69.0–85.0	56	70	60.0–81.3	88	81	73.8–85.6
BMI [kg/m²]	144	26	22.7–27.6	56	24.9	21.3–28.9	88	25.5	23.5–27.5
Gender [female]	147	57 (38.8 %)		57	—		90	—	
Immunotherapy cycles									
s/p 1 cycle	147	10 (6.8 %)		57	3 (5.3 %)		90	7 (7.8 %)	
s/p 2 cycle		25 (17.0 %)			7 (12.3 %)			18 (20.0 %)	
s/p 3 cycle		23 (15.6 %)			9 (15.8 %)			14 (15.6 %)	
s/p 4 cycle		89 (60.5 %)			38 (66.7 %)			51 (56.7 %)	
Distant Metastases [in > 1 visceral organ]	147	50 (34.7%)		57	14 (24.6%)		90	36 (40.0 %)	
**Blood-based markers**									
LDH [> 248 U/l]	131	51 (38.9%)		49	22 (44.9 %)		82	29 (35.4 %)	
S100B [> 0.11 µg/l]	94	47 (50.0 %)		36	15 (41.7%)		58	32 (55.2 %)	
**Macroangiopathy**									
Hard Plaque Volume [0.1 cm^3^]	147	2.4	0.1–14.9	57	1.2	0.0–6.8	90	4.3	0.2–26.5
Soft Plaque Volume [mm^3^]	147	52.7	0.0–218.9	57	20	0.0–118.9	90	63.4	0.0–305.4
Total Plaque Index [mm^2^]	147	2.3	0.2–13.7	57	1.3	0.1–6.2	90	5	0.4–21.2
**Microangiopathy**									
Fazekas Score for dWMH									
FS 0	144	30 (22.8%)		56	8 (14.2%)		88	22 (25.0%)	
FS 1		82 (56.9 %)			37 (66.1 %)			45 (51.1 %)	
FS 2		28 (19.4 %)			8 (14.3 %)			20 (22.7 %)	
FS 3		4 (2.8 %)			3 (5.6 %)			1 (1.1 %)	
**Muscle mass**									
Total Psoas Area [cm²]	147	7.5	6.1–9.1	57	6.1	5.2–6.7	90	8.7	7.5–10.2
Mean Psoas Density [10 HU]	147	5	4.5–5.3	57	5.1	4.6–5.4	90	4.9	4.4–5.3
**Fat tissue**									
Total Body Fat [cm²]	147	109.5	82.8–147.4	57	105.7	73.8–178.4	90	110.2	88.5–137.1
S.c. Adipose Tissue [cm²]	147	64.3	48.3–90.0	57	74.3	51.3–105.6	90	60.4	48.2–72.7
Visceral Adipose Tissue [cm²]	147	44.6	26.9–66.7	57	34.1	18.6–51.6	90	47.4	33.2–70.2

Abbrev.: n, number of patients; IQR, interquartile range; No., number; s.c., subcutanous; LDH, lactate dehydrogenase; dWMH, deep white matter hyperintensities; HU, Hounsfield Units; FS, Fazekas Score; s/p, status post.

### Macroangiopathy

**Figure 1:**
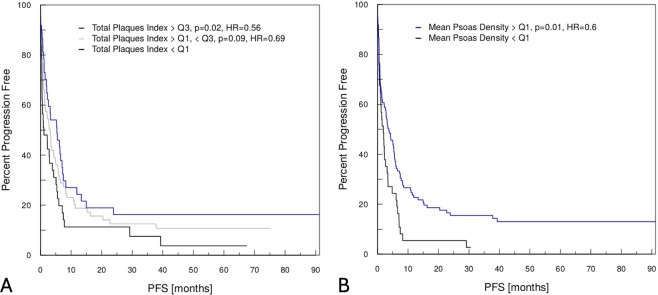
Kaplan-Meier survival curves of (**A**) the total plaque index (TPI) and (**B**) the mean psoas density (MPD). **(A)** The total plaque index (TPI) was defined as the body height adjusted sum of the hard and soft plaque volume of the infrarenal aorta. Patients with a TPI in the upper quartile Q3 (blue) had nearly half the risk of disease progression at every time point compared to patients with a TPI in the lower quartile Q1 (black) (univariate Cox regression analysis, p = 0.02, hazard ratio (HR) 0.56, confidence interval (CI) 0.35–0.92). Patients with a TPI in the interquartile range are outlined in grey color and display an intermediate risk for disease progression (univariate analysis, p = 0.09, HR 0.69, CI 0.46–1.06). **(B)** The mean psoas density (MPD) was measured on the fourth lumbar vertebrae level on both sides and averaged. Patients with an MPD greater than Q1 (blue) had a 40% reduced risk of disease progression compared to patients with an MPD in the lower quartile (black) (univariate Cox regression analysis, p = 0.01, HR 0.60, CI 0.40–0.88).

Body height correction of the total plaque volume defined as the sum of hard and soft plaque volume was performed via calculating the ratio of the total plaque volume and the anterior height of the os sacrum (hereinafter referred to as total plaque index (TPI)). Median TPI (IQR) was 2.3 (0.2–13.7) mm² and TPI correlated significantly with PFS in univariate Cox regression analysis with a reduction of progression risk by 9% every 10 mm² (p_univ_ = 0.04, HR 0.91, CI 0.84–1.00). Patients with a TPI in the upper quartile had approximately half of the progression risk at any time point compared to patients with a TPI in the first quartile (p_univ_ = 0.02, HR 0.56, CI 0.35–0.92, Fig. 1A). Median volume (IQR) of hard and soft plaques was 2.4 (0.1–14.9) cm³/10 and 52.7 (0.0–218.9) mm³, respectively. Neither hard nor soft plaque volume alone correlated significantly with PFS in univariate analysis (p_univ_ = 0.06 and p = 0.12, respectively). Detailed results including HRs and CIs of both uni- and multivariate analysis are presented in Table 2.

**Table 2:** Results of uni- and multivariate Cox regression analysis.

Parameter	n	Univariate Cox regression			n	Multivariate Cox regression		
		p-value	HR	95%-CI		p-value	HR	95%-CI
**Macroangiopathy**								
Hard Plaque Volume [cm³, range: 0.0–20.6]	147	0.06	0.95	0.89–1.00	—	—	—	—
Soft Plaque Volume [100 mm³, range: 0.0–34.7]	147	0.12	0.96	0.91–1.01	—	—	—	—
Total Plaque Index [10 mm², range 0.0–15.8]	147	0.04	0.91	0.84–1.00	147	0.03	0.88	0.79–0.99
Total Plaque Index								
≤Q1	37	ref	ref	ref	—	—	—	—
Q1<TPI<Q3	73	0.09	0.69	0.46–1.06	—	—	—	—
≥Q3	37	0.02	0.56	0.35–0.92	—	—	—	—
**Microangiopathy**								
Fazekas Score for dWMH [0–3]								
≤1	112	ref	ref	ref	—	—	—	—
>1	32	0.92	0.98	0.65–1.47	—	—	—	—
**Muscle mass**								
Total Psoas Area [cm², range: 2.0–14.0]								
Total cohort	147	0.27	0.96	0.89–1.03	—	—	—	—
Men	90	0.25	0.94	0.85–1.05	—	—	—	—
Women	57	0.73	1.04	0.84–1.29	—	—	—	—
Mean Psoas Density [10 HU, range: 2.6-6.3]	147	0.01	0.71	0.55–0.93	147	0.001	0.63	0.49–0.83
Mean Psoas Density [HU]								
≤Q1	37	ref	ref	ref	—	—	—	—
>Q1	110	0.01	0.6	0.40–0.88	—	—	—	—
**Fat tissue**								
Total Body Fat [10 cm², range_total_: 2.6–27.8]								
Total cohort	147	0.8	1	0.97–1.03	—	—	—	—
Men	90	0.76	1.01	0.97–1.05	—	—	—	—
Women	57	0.8	1.01	0.96–1.05	—	—	—	—
S.c. Adipose Tissue [10 cm², range_total_: 1.4–18.8]								
Total cohort	147	0.7	1	1.00–1.00	—	—	—	—
Men	90	0.64	1	1.00–1.00	—	—	—	—
Women	57	1	1	1.00–1.00	—	—	—	—
Visceral Adipose Tissue[10 cm², range_total_: 0.9–13.8]								
Total cohort	147	0.98	1	0.94–1.06	—	—	—	—
Men	90	0.94	1	0.93–1.08	—	—	—	—
Women	57	0.51	1.04	0.93–1.16	—	—	—	—
**Blood-based markers**								
LDH [>248 U/l]	131	0.13	1.34	0.92–1.95	131	0.39	1.18	0.81–1.74
S100B [>0.11 µg/l]	94	0.03	1.63	1.05–2.55	94	0.05	1.57	1.00–2.47
**Clinical parameter**								
No. immunotherapy cycles [s/p 4 ipilimumab cycles]	147	0.18	0.79	0.55–1.12	147	0.18	0.78	0.55–1.12
Gender [male]	147	0.47	0.88	0.61–1.25	147	0.5	0.88	0.60–1.28
Age [years]	147	0.25	0.99	0.98–1.01	147	0.5	0.99	0.98–1.01
Distant Metastases [> 1 visceral organ]	147	0.07	1.39	0.97–2.00	147	0.01	1.61	1.10–2.37
Weight [kg]								
Total cohort	144	0.84	1	0.99–1.01	—	—	—	—
Men	88	0.78	1	0.99–1.02	—	—	—	—
Women	56	0.65	1	0.99–1.02	—	—	—	—
Body Mass Index [kg/m²]								
Total cohort	144	0.64	1.01	0.97–1.05	—	—	—	—
Men	88	0.95	1	0.95–1.06	—	—	—	—
Women	56	0.48	1.02	0.97–1.07	—	—	—	—

Abbrev.: HR, Hazard ratio; CI, confidence intervals; No., number; n, number of patients, s.c., subcutanous; LDH, lactate dehydrogenase; dWMH, deep white matter hyperintensities; HU, Hounsfield Units; s/p, status post.

### Microangiopathy

For 144 of 147 patients (98.0%) a cranial MRI with a fluid-attenuated inversion recovery sequence (FLAIR) was available in the corresponding time period. Thirty of 144 patients (22.8%) did not show any deep white matter hyperintensties (dWMHs) (Fazekas score (FS) = 0), whereas 82 of 144 patients (56.9%) depicted punctate foci of dWMHs (FS 1), 28 of 144 patients (19.4%) showed beginning confluence of dWMHs (FS 2) and only 4 of 144 patients (2.8%) displayed severe presence of T2 hyperintense lesions (FS 3) i.e. large confluent areas. The FS did not correlate with PFS in univariate Cox regression analysis, when comparing patients with a FS > 1 (n = 32) with those with a FS ≤ 1 (n = 112, p_univ_ = 0.92, HR 0.98, CI 0.65–1.47).

### Muscle mass

Median mean psoas density (MPD, (IQR)) was 49.9 (45.0–52.9) Hounsfield Units (HU). MPD correlated significantly with PFS in univariate regression analysis with a reduction of progression risk of around 30% every 10 HU (p_univ_ = 0.01, HR 0.71, CI 0.55–0.93). Furthermore, patients with an MPD greater than the lower quartile Q1 had a 0.6-fold reduced progression risk at any time point compared to patients in the lowest quartile of the psoas density, i.e. patients with the highest fatty infiltration of the psoas muscle (p_univ_ = 0.01, HR 0.6, CI 0.40–0.88, Fig. 1B).

Median total psoas area (IQR) was 7.5 (IQR 6.1–9.1) cm². Total psoas area was not significantly associated with PFS in univariate analysis even after stratification by gender (p_univ_ ≥0.25 each, for HRs and CIs see Table 2).

### Adipose tissue

Median total body fat (IQR) was 109.5 (82.8–147.4) cm²; median visceral adipose tissue area (IQR) was 44.6 (26.9–66.7) cm² and median subcutaneous adipose tissue area (IQR) was 64.3 (48.3–90.0) cm². For calculation of the body mass index (BMI) four patients had to be excluded due to missing data. Median body weight (IQR) was 78.0 (60.0–85.0) kg and median BMI (IQR) was 26.0 (22.7–27.6) kg/m². No adipose tissue parameter correlated significantly with PFS, even after separate assessment of male and female patients (p > 0.05, for HRs and CIs see Table 2).

### Blood-based parameters

In 131 patients (89.1%) serum LDH had been quantified within three months prior to immunotherapy with 51 of the patients revealing an increased serum LDH level (38.9%). Univariate Cox regression analysis did show a trend to increase of progression risk in patients with an elevated serum LDH prior to immunotherapy (p_univ_ = 0.13, HR 1.34, CI 0.92–1.95).

S100B value was available in 94 patients (63.9%) with 47 (50.0%) patients showing an increased S100B value before initiation of ipilimumab therapy. S100B value was significantly associated with PFS in univariate analysis (p_univ_ = 0.03, HR 1.63, CI 1.05–2.55).

### Multivariate Cox regression analysis

Finally, multivariate analysis was conducted including all metrics with p_uni_ <0.2, i.e. MPD, TPI, age, gender, number of immunotherapy cycles and extent of distant metastases. Multivariate analysis confirmed the significant impact of the MPD and TPI on PFS (MPD: p_multi_ = 0.001, HR 0.66, CI 0.51–0.85; TPI: p_multi_ = 0.03, HR 0.99, CI 0.98–1.00). Furthermore, patients with distant metastases in more than one visceral organ had a significantly shorter PFS than patients with metastases in only one visceral organ ± cutaneous or nodal metastases (p_multi_ = 0.02, HR 1.56, CI 1.06–2.30).

After adjustment for age, gender, distant metastases and immunotherapy cycles, increased S100B level revealed a close to significant trend to decreased PFS (p_multi_ = 0.05, HR 1.57, CI 1.00–2.47, n = 94), while increased serum LDH was not significantly associated with PFS (p_multi_ = 0.39, HR 1.18, CI 0.81–1.74, n = 131). Results of multivariate analysis are presented in Table 2.

## Discussion

In the current study, we investigated the value of different body composition measures to predict response to immunotherapy with ipilimumab. Apart from confirming mean psoas density, a surrogate for sarcopenia, as a positive predictive factor for outcome to immunotherapy, we report a surprising positive correlation between total plaque burden of the abdominal aorta and progression free survival.

Even though the immune system is generally capable not just to identify, but also to eradicate neoplastic cells, it is physiologically impeded by immune check points in order to prevent autoimmunity. Antibodies that block the checkpoint CTLA-4 or PD1/PD-L1 have demonstrated efficacy in a number of malignancies. However, response rates are variable and these novel therapies, especially those relying on CTLA-blockade and thereby targeting an early point in anti-tumoral immune response, bear a significant risk of immune-related adverse events (irAE) with clinically detectable irAEs in up to 72% and grade 3–4 irAEs in 24% of patients treated with ipilimumab^24,25^.

Beside the potential irAE, therapy with checkpoint inhibitors is very cost-intensive with an ipilimumab monotherapy amounting to approximately $160,000 per patient and combined ipilimumab and nivolumab therapy to $300,000^26^. Both potential irAE and high costs result in an urgent demand for predictive biomarkers that allow for reliable identification of patients that are likely to benefit from immunotherapy.

Our results confirm the validity of the sarcopenia-frailty concept, that predicts a correlation between sarcopenia and disease outcome, even in patients undergoing immunotherapy^17^. The prognostic or predictive value of the psoas density was shown in a variety of other pathological conditions before, such as trauma, liver transplantation as well as outcome after tumor resection or cardiovascular surgery^27–31^.

Furthermore, we assessed the potential of vessel health in predicting response to immunotherapy and thus defined the total plaque index, i.e. the body height corrected sum of the hard and soft plaque volume, to quantify extent of macroangiopathy. Body height correction was performed to rule out any influence of the body height on the plaque volume, as it seemed reasonable that greater vessel wall surface might correlate with greater plaques burden.

As sacrococcygeal dimensions should be used for body height estimation when long bones are not available, body height correction was performed via normalizing the plaque volume by the total anterior height of the sacral and coccygeal vertebrae as previously described^32,33^. This measurement had not just given the most accurate stature estimate in forensic studies, but is also easy to perform and routinely included in the examination area.

The shown positive correlation between TPI and PFS might reveal a surprising hint towards pathophysiology of immune checkpoint blockade: While the positive relationship between macroangiopathy and immune status may appear paradoxical in the first instance, it seems conceivable that systemic inflammatory diseases such as atherosclerosis enhance the effect of immunotherapy due to a generally increased immune activity. This would be in accordance with the finding that patients with a tendency to autoimmunity and irAE are more likely to benefit from immune checkpoint blockade and that premature interruption of therapy due to irAEs is known to not having a negative effect on progression free survival^34–37^.

Notably, the isolated assessment of the hard plaque volume did not reveal a significant correlation with response to immunotherapy, whereas consideration of the soft plaques volume, which represents only around one tenth of the total plaque burden, revealed a significant impact on PFS. This might be due to the pronounced inflammatory component of noncalcified atheroma whose progress is known to be induced by immune cells and T-cell derived cytokines and whose stability negatively correlates with levels of acute-phase proteins such as c-reactive protein or serum amyloid A^38,39^.

However, even though cerebral small vessel disease as well is said to be associated with chronic inflammation to some degree^40^, Fazekas Score for deep white matter lesions, i.e. a surrogate of microangiopathy, did not correlate with progression free survival in our study.

Furthermore, fat tissue volume has previously been discussed to have an either negative effect on outcome after immunotherapy due to higher comorbidity or otherwise a positive effect due to its known endocrine function. However, our study did not reveal any impact of adipose tissue volume on PFS and therewith contradicts both the results of Sabel *et al*. who reported on a negative impact of increasing visceral fat distance on overall survival after treatment with ipilimumab in 44 patients^17^ and the findings of McQuade *et al*. who reported on a positive correlation between BMI and therapy outcome in male patients treated with either targeted or immunotherapy^19^.

Compared to the multiple tissue and blood-based biomarkers that have been under investigation so far such as tumor PD-L1 expression, neutrophil to lymphocyte ratio, absolute lymphocyte count, level of lactate dehydrogenase, S100B or density of tumor infiltrating lymphocytes^15,41–43^, the imaging derived biomarkers investigated in this study have the practical advantage that they occur in routine clinical practice and do not require any additional examinations or blood sampling. Moreover, morphometric assessment demands very little time with results being available instantly, prior to a potential start of therapy and without the need of follow-ups for kinetic analysis.

Furthermore, prognostic equivalency of the morphometric parameters compared to the assessed blood-based markers was proven with total plaque index and mean psoas density correlating significantly with PFS, while increased S100B level prior to start of immunotherapy did only show a borderline effect on PFS and elevated serum LDH was not associated with PFS at all. - Not to forget that hazard ratios depend on the subdivision and range of the explanatory variable and therefore must not be compared between the different metrics one-by-one…

Our study has limitations: CT data analysis was conducted semi-automatically with consecutive increase of expenditure of time as well as reduction of reproducibility. Furthermore, our analysis was limited to patients with malignant melanoma and treatment with ipilimumab even though inclusion of patients with different cancer entities and therapy with other checkpoint inhibitors or different immunotherapy regimes would have been desirable.

In conclusion, our study reveals a positive correlation between manifestation of macroangiopathy and response to immunotherapy, which might be exploited as an easily performed predictive imaging biomarker for outcome to immunotherapy. Furthermore, our study results include valuable hints towards pathophysiology and demand for a deeper understanding of the association of chronic inflammatory disease and immune status.

## Patients and Methods

### Patients and ethics

The ethics committee of the University of Heidelberg approved this study and written informed consent was waived due to its retrospective character. The study was conducted in accordance with all relevant guidelines and regulations.

We reviewed the medical record and imaging studies of all patients who were referred to our imaging center between August 2010 and April 2017 with a diagnosis of malignant melanoma. Patients with ipilimumab therapy were included in the study and abdominal CT scans and cerebral MRI performed up to 3 months prior to or after start of immunotherapy were analyzed. Patients without available CT of the abdomen and patients who had received more than four cycles of immunotherapy were excluded (n = 8). Patients without available FLAIR sequence in the aforementioned period were not included in the analysis of deep white matter hyperintensities (n = 3).

### Image and data analysis

CT scans were performed at our institution on a dual-energy Siemens system (Somatom Definition Flash, Siemens). Coronal and axial plane of a venous post-contrast CT (Imeron 300 M) of the abdomen were pseudonymized and transferred to the Medical Imaging Interaction Toolkit (MITK, version 2016.03.0).

The majority of cranial FLAIR MRI was performed at our institution on a 1.5 Tesla MRI system (Magnetom Symphony, Siemens) with the following sequence parameters: echo time 98 ms, repetition time 8000 ms, inversion time 2340 ms, slice thickness 5 mm, field of view 230, number of slices 30, flip angle 150°, acquisition matrix 0/320/205/0, number of averages 2 and acquisition time 5:38 min. If no cMRI with a FLAIR sequence of our institution, but of another institution was available for the corresponding period, external imaging was consulted for image analysis.

**Figure 2:**
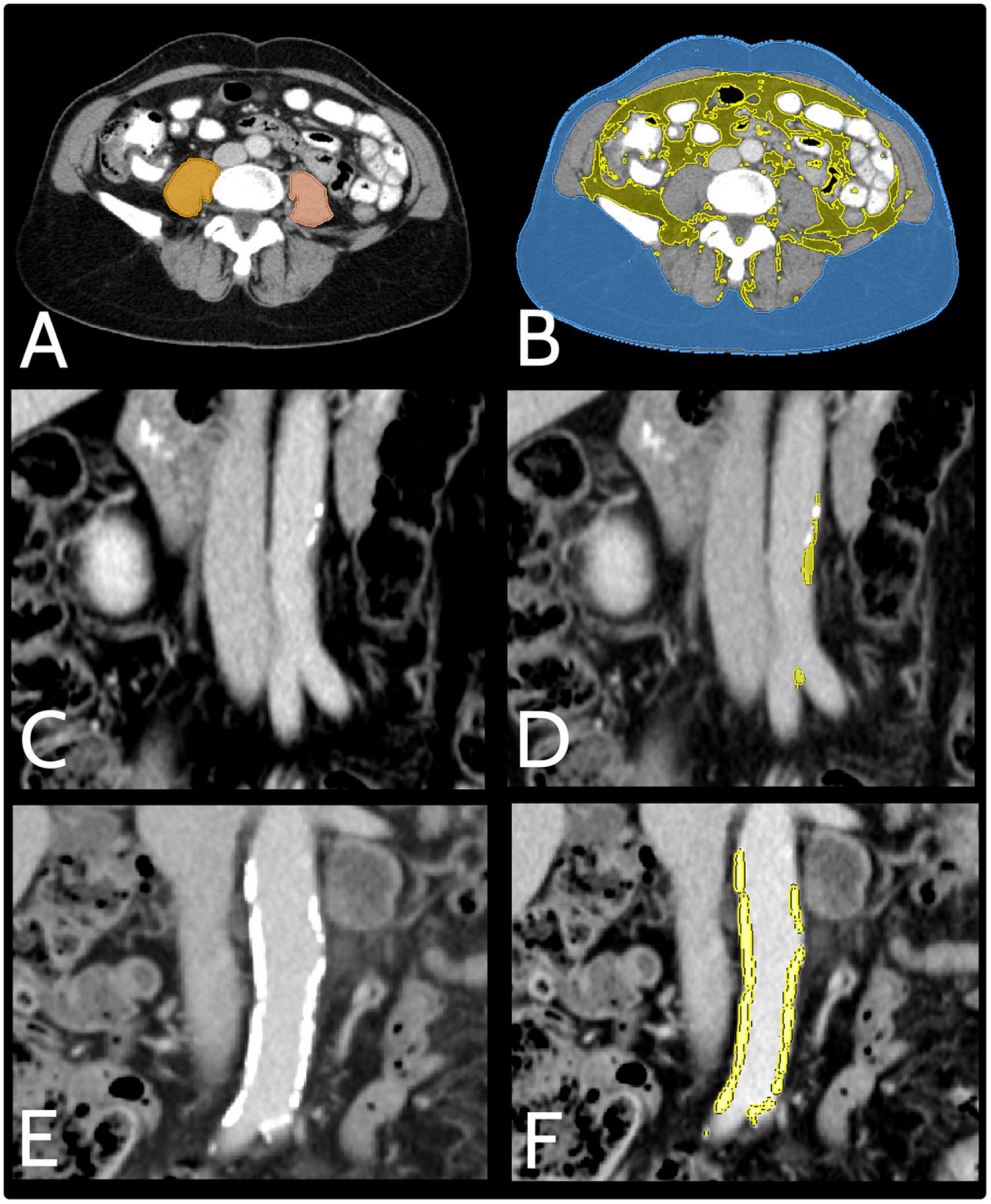
Example of applied image analysis. Portal-venous CT scans of the abdomen of a (**A**, **B**) 56-year-old female, (**C**,** D**) 64-year-old male and (**E, F**) 67-year-old male with malignant melanoma prior to immunotherapy with ipilimumab. **(A)** Semi-automatic segmentation of the psoas muscles in the axial plane at the L4 level for calculation of the mean psoas area and the mean psoas density. **(B)** Semi-automatic segmentation of the visceral and subcutaneous adipose tissue area in the axial plane at the L4 level with fat density thresholds set to −190 HU to −30 HU. **(C–F)** Three-dimensional segmentation of the soft (**D**) and hard (**F**) plaque volume compared to the non-segmented images (**C**,** E**). Mean applied density thresholds were 100 (range 60–100) HU and 185 (180–220) HU for soft and hard plaques, respectively.

**Figure 3:**
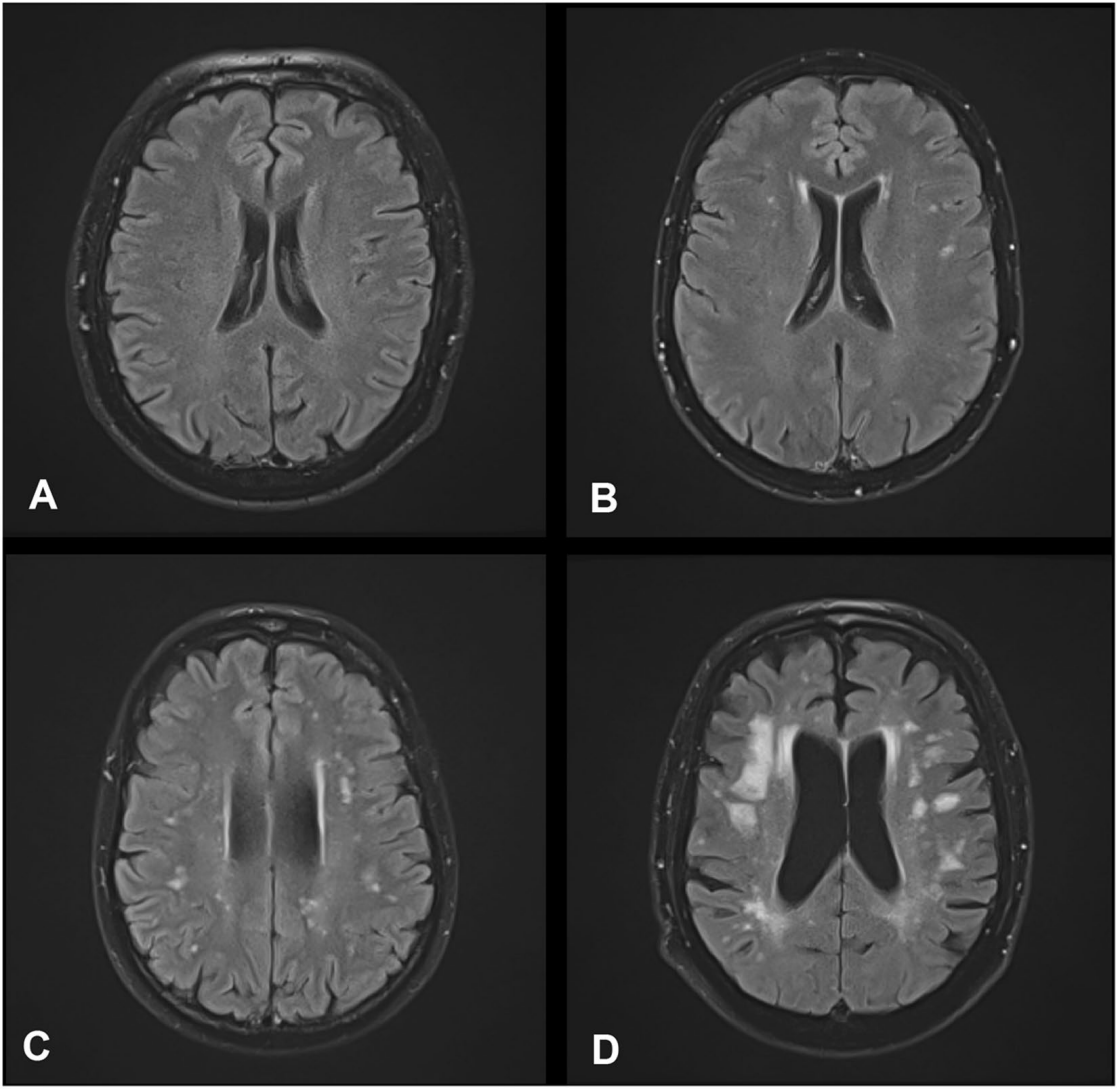
Example of the applied Fazekas scoring system (FS) for quantification of deep white matter hyperintensities (dWMH) on axial fluid-attenuated inversion recovery MRI (FLAIR). (**A**) Absence of hyperintense lesions in the deep white matter on FLAIR MRI (FS 0). (**B**) Punctate hyperintense foci of dWMHs on FLAIR MRI (FS 1). (**C**) Beginning confluence of dWMHs on FLAIR MRI (FS 2). (**D**) Large confluent areas of hyperintense lesions on FLAIR MRI (FS 3).

Image analysis was carried out with the reader blinded to the patients’ data and according to the following description (for an example of the applied image post processing see Fig. 2):

- Degree of macroangiopathy was determined for the abdominal aorta inferior to the renal arteries and superior to the bifurcations of the common iliac arteries into the external and internal iliac arteries. The aorta was semi-automatically segmented on the coronal plane and the volume of hard and soft plaques was determined via thresholding with a median threshold of 185 (range 180–220) HU for calcifications and 100 (range 60–100) HU for soft plaques^44^, respectively. Segmentations were corrected manually if necessary.
- Psoas muscle contours were drawn on the axial plane by using the live wire tool on the fourth lumbar vertebrae (L4) level, and total psoas areas as well as MPD were calculated.
- The ventral length of the sacrum was measured from the promontorium to the inferior coccyx with the help of the curve tool on the median sagittal plane, which was reconstructed by MITK from the axial plane.
- For assessing the fatty tissue area, fat density thresholds of −190 to −30 HU were used. The visceral adipose tissue area was defined as the total area inside the abdominal fascia meeting fat-density thresholds, whereas the subcutaneous adipose tissue area was defined as the total area between the abdominal fascia and skin on L4 level. Total body fat was defined as the sum of subcutaneous and visceral adipose tissue area.
- Finally, the FLAIR sequence was used to determine the degree of deep white matter hyperintensitie, which are attributed to chronic small vessel disease, by applying the Fazekas Scoring System (Fig. 3)^45,46^. As the aetiology of deep and periventricular white matter hyperintensities differ, the reader was instructed to disregard hyperintensities associated with the ventricles.

Additionally, patient height and weight were extracted from the medical records to calculate their BMI. Furthermore, serum LDH and S100B values quantified within three months prior to immunotherapy as well as number of sites with distant metastases were determined. Progression free survival was calculated as the time interval between the last immunotherapy cycle and change in therapy due to disease progression or date of death. However, for patients who did not experience disease progression in the study period, PFS was calculated with the date of the last medical report with documented stable disease.

### Statistical analysis

Descriptive statistics were carried out using Microsoft Excel 2016 and statistical testing was performed with the software package R (R Foundation for Statistical Computing, version 3.4.4, 2018–03–15). Significance was set at the p < 0.05 level. To control for multiple testing, a hierarchal hypothesis testing approach was performed: Firstly, univariate Cox regression analysis was performed as screening for potential predictive factors. Subsequently, multivariate analysis was conducted including known potential confounders such as gender and age as well as all parameters with p_univ_ < 0.02 to confirm their independent predictive value and to compare the degree of impact of the individual parameters.

## Data Availability

All data is available from the corresponding author on reasonable request in pseudonymised form.
